# Antenatal management and maternal/fetal outcomes associated with hyperglycaemia in pregnancy (HIP) in Uganda; a prospective cohort study

**DOI:** 10.1186/s12884-021-03795-5

**Published:** 2021-05-19

**Authors:** Jack Milln, Betty Nakabuye, Barnabas Kahiira Natamba, Isaac Sekitoleko, Michael Mubiru, Arthur Araali Namara, Samuel Tumwesigire, Salome Tino, Mandy Mirembe, Ayoub Kakande, Brian Agaba, Faridah Nansubuga, Daniel Zaake, Ben Ayiko, Herbert Kalema, Sarah Nakubulwa, Musa Sekikubo, Annettee Nakimuli, Emily L. Webb, Moffat J. Nyirenda

**Affiliations:** 1 Non-Communicable Diseases Theme, Medical Research Council/Uganda Virus Research Institute and London School of Hygiene and Tropical Medicine (MRC/UVRI & LSHTM) Uganda Research Unit, Plot 51-59, Nakiwogo Road, P. O. BOX 49, Entebbe, Uganda; 2 Department of Endocrinology and Diabetes, Queen Mary University of London, Mile End Road, London, UK; 3 Rubaga Uganda Martyrs Hospital, Kampala, Uganda; 4 School of Public Health, Makerere University College of Health Sciences, Kampala, Uganda; 5 St. Francis Hospital, Nsambya, Kampala, Uganda; 6 Entebbe Regional Referral Hospital, Entebbe, Uganda; 7 Masaka Regional Referral Hospital, Masaka, Uganda; 8 Department of Obstetrics and Gynaecology, School of Medicine, Makerere University College of Health Sciences, Kampala, Uganda; 9 Kawempe National Referral Hospital, Kampala, Uganda; 10 London School of Hygiene and Tropical Medicine (LSHTM), London, UK

**Keywords:** Pregnancy, Diabetes, Gestational, Sub Saharan-Africa, Macrosomia

## Abstract

**Background:**

Hyperglycaemia in pregnancy (HIP) is associated with complications for both mother and baby. The prevalence of the condition is likely to increase across Africa as the continent undergoes a rapid demographic transition. However, little is known about the management and pregnancy outcomes associated with HIP in the region, particularly less severe forms of hyperglycaemia. It is therefore important to generate local data so that resources may be distributed effectively. The aim of this study was to describe the antenatal management and maternal/fetal outcomes associated with HIP in Ugandan women.

**Methods:**

A prospective cohort study of 2917 pregnant women in five major hospitals in urban/semi-urban central Uganda. Women were screened with oral glucose tolerance test (OGTT) at 24–28 weeks of gestation. Cases of gestational diabetes (GDM) and diabetes in pregnancy (DIP) were identified (WHO 2013 diagnostic criteria) and received standard care. Data was collected on maternal demographics, anthropometrics, antenatal management, umbilical cord c-peptide levels, and pregnancy outcomes.

**Results:**

Two hundred and seventy-six women were diagnosed with HIP (237 classified as GDM and 39 DIP). Women had between one and four fasting capillary blood glucose checks during third trimester. All received lifestyle advice, one quarter (69/276) received metformin therapy, and one woman received insulin. HIP was associated with large birthweight (unadjusted relative risk 1.30, 95% CI 1.00–1.68), Caesarean delivery (RR 1.34, 95% CI 1.14–1.57) and neonatal hypoglycaemia (RR 4.37, 95% CI 1.36–14.1), but not perinatal mortality or preterm birth. Pregnancy outcomes were generally worse for women with DIP compared with GDM.

**Conclusion:**

HIP is associated with significant adverse pregnancy outcomes in this population, particularly overt diabetes in pregnancy. However pregnancy outcomes in women with milder forms of hyperglycaemia are similar to those with normoglycaemic pregnancies. Intervention strategies are required to improve current monitoring and management practice, and more research needed to understand if this is a cost-effective way of preventing poor perinatal outcomes.

**Supplementary Information:**

The online version contains supplementary material available at 10.1186/s12884-021-03795-5.

## Introduction

Hyperglycaemia first detected in pregnancy (HIP) is a common condition associated with significant adverse pregnancy outcomes. HIP is commonly diagnosed through oral glucose tolerance testing (OGTT), and is classified as overt diabetes in pregnancy (DIP) or gestational diabetes mellitus (GDM) when hyperglycaemia is less severe [1]. While there is a clear association between DIP and adverse pregnancy outcome, the impacts of hyperglycaemia in the GDM range (fasting glucose 5.1–7.0 mmol/L, 2-h OGTT glucose level 8.5–11.0 mmol/L) has been more controversial. Recently, however, the HAPO study showed hyperglycaemia within the GDM range was linearly associated with adverse pregnancy outcomes, notably large birthweight (> 90th centile) [2]. This has led to recent tightening of international diagnostic criteria for GDM in order to capture women with milder derangements in glucose control [3], though implementation of these criteria may vary at a local level, even within countries. Subsequently, some studies have shown that treating such mild levels hyperglycaemia is associated with modest improvement in outcomes, although in most cases this required intensive interventions such as insulin use, multiple daily self-monitoring of blood glucose or induction of labour [4, 5].

The International Diabetes Federation (IDF) estimates that 1 in 6 women in the African region may be affected by hyperglycaemia in pregnancy, raising the profile of HIP on the international development agenda around non-communicable diseases (NCD) prevention and management [6]. The prevalence is set to further increase as the continent continues to undergo a rapid demographic and nutritional shift associated with urbanisation. Despite this, screening and treatment of HIP is not common in most countries in sub-Saharan Africa (SSA), and there is paucity of studies on screening, treatment and obstetric outcomes of HIP; loose recommendations are largely based on external evidence, or on small studies with heterogeneous methodologies and criteria [7–9]. However, in most cases, screening and treatment methods may not be appropriate and are not followed, commonly due to resource constraints. Importantly, both the HAPO and subsequent intervention studies were largely undertaken in high-income countries, and the evidence may not necessarily directly translate to populations in SSA. This necessitates rigorous local research to develop optimal screening and management strategies that will identify and target women with HIP who are at significant risk of clinically relevant adverse obstetric outcomes.

In this study, we used a cohort of well characterised women with and without HIP in Uganda to describe how HIP is managed, and the frequency of adverse outcomes (particularly large birthweight and perinatal death) associated with this condition in this population.

## Methods

### Setting

This observational cohort study recruited women attending antenatal care at five major hospitals in urban and peri-urban areas of central Uganda between 13th June 2018 and 31st October 2019. Three are public facilities managed by the Uganda Ministry of Health, and two are private-not-for-profit hospitals managed by the Uganda Catholic Medical Services Bureau.

### Participants

Pregnant women were eligible to participate if they were 18 years or older and between 24 and 28 weeks of gestation calculated using date of last menstrual period and/or earliest obstetric ultrasound scan where available. Women were excluded if they had one or more of the following exclusion criteria: known diagnosis of diabetes, significant medical comorbidity (such as heart failure, renal disease, severe anaemia), multiple pregnancy, inability to provide informed consent, or plans to deliver at a non-study facility.

Women were approached in the antenatal clinic by the research team and screened for inclusion and exclusion criteria.

At recruitment, standardised questionnaires were used to collect data on socio-demographic and lifestyle factors (including age, level of education, smoking status and alcohol use). Questionnaires also covered family, medical (including HIV status) and reproductive history (parity, gravidity and complications in prior pregnancies). Weight and height were measured using calibrated Seca scales and stadiometers. After 30 min of rest, three seated blood pressure measurements, with 5 min rest in between, were collected on the right arm using portable sphygmomanometers (OMRON-Healthcare-Co HEM-7211-E-Model-M6; Kyoto, Japan). We used the mean of the last two blood pressure readings.

### Oral glucose tolerance test

Participants underwent a standard oral glucose tolerance test after an overnight fast of at least 8 h. A fasting venous blood glucose was collected, and participants were then given 82.5 g glucose monohydrate (equivalent to 75 g anhydrous glucose) dissolved in 250 ml of water. Repeat venous blood samples were taken at 60 and 120 min. Samples were immediately centrifuged at study sites and plasma stored on ice. All samples were analysed centrally at the MRC/UVRI and LSHTM Clinical and Diagnostics Laboratory in Entebbe, within 4 h of collection, or stored at − 80 °C for subsequent analysis.

### Diagnosis and management of women with hyperglycaemia in pregnancy

HIP was diagnosed according to WHO 2013 criteria as GDM: fasting glucose ≥5.1 and < 7.0 mmol/L or 1-h glucose ≥10.0 mmol/L or 2-h glucose ≥8.5 and < 11.1 mmol/L; and, DIP: fasting glucose ≥7.0 mmol/L or 2-h glucose ≥11.1 mmol/L. Women with hyperglycaemia in pregnancy were notified and invited to meet the local obstetric team for further management. A summary of local management practices conducted prior to the study is provided in the Supplementary Appendix (Table S1). Clinicians were provided with a basic treatment protocol based on the FIGO pragmatic guide for diabetes antenatal care in the resource-limited setting [10]. Antenatal management was recorded with a standardised proforma by the obstetric team at each study site including the number of antenatal visits, fasting capillary glucose values, treatment administered, and third trimester ultrasound scan results. For this study, we considered HIP as ‘controlled’, ‘partially controlled’ or ‘uncontrolled’ if the mean of the two fasting capillary blood glucose values prior to delivery were < 5.1 mmol/L, 5.1–7.0 mmol/L, or > 7.0 mmol/L respectively. If cases were not seen in the antenatal clinic or only had one fasting capillary glucose result, they were coded as ‘unknown’.

### Collection of outcome data

Maternal and neonatal outcomes were extracted from mothers’ records at the time of delivery and recorded by midwives. These included: maternal antenatal complications (hypertensive disorders of pregnancy, poly/oligohydramnios), delivery complications (prolonged labour, ruptured uterus, shoulder dystocia), mode of delivery, birthweight and gestational age of neonate. Hypertensive disorders of pregnancy included gestational hypertension, pre-eclampsia, and eclampsia. Macrosomia and low-birthweight were defined as birthweight > 4 kg and < 2.5 kg, respectively. Further data were recorded at the time of discharge from hospital and included neonatal complications (neonatal admission, hypoglycaemia, jaundice) and neonatal death. Neonatal admission was defined as formal admission of the neonate to the special baby unit for observation and/or treatment beyond routine neonatal care. Umbilical cord blood, to estimate serum c-peptide concentration, was obtained at the time of delivery by midwives; samples were immediately centrifuged at study sites, stored on ice, and analysed at the MRC/UVRI & LSHTM Uganda Research Unit central laboratory.

### Outcomes of interest

The primary outcomes of interest were birthweight > 90th centile using the INTERGROWTH-21 population standards [11] and perinatal death (stillbirth > 24 weeks and neonatal death < 28 days). Other outcomes of interest were Caesarean delivery, preterm birth (< 37 weeks), and neonatal admission.

### Statistical analysis

Participants’ baseline characteristics were summarised using means, medians, standard deviations, and interquartile ranges for continuous variables where applicable. Categorical data were summarized using numbers and proportions. We used Pearson’s chi-square and Fisher’s exact tests to assess the associations between HIP and maternal and neonatal outcomes, and present crude risk ratios (RRs) with 95% confidence intervals (95% CI).

All analyses were conducted in STATA 15.1 (College Station, Texas).

### Ethical approval

This research project was approved by the research and ethics committee of the Uganda Virus Research Institute (approval GC/127/19/04/625) and the Uganda National Council for Science and Technology (approval HS2340). All participating women gave informed written consent. A minimal compensation for participants’ time and meal after undergoing the OGTT was provided.

## Results

### Maternal characteristics

**Fig. 1:**
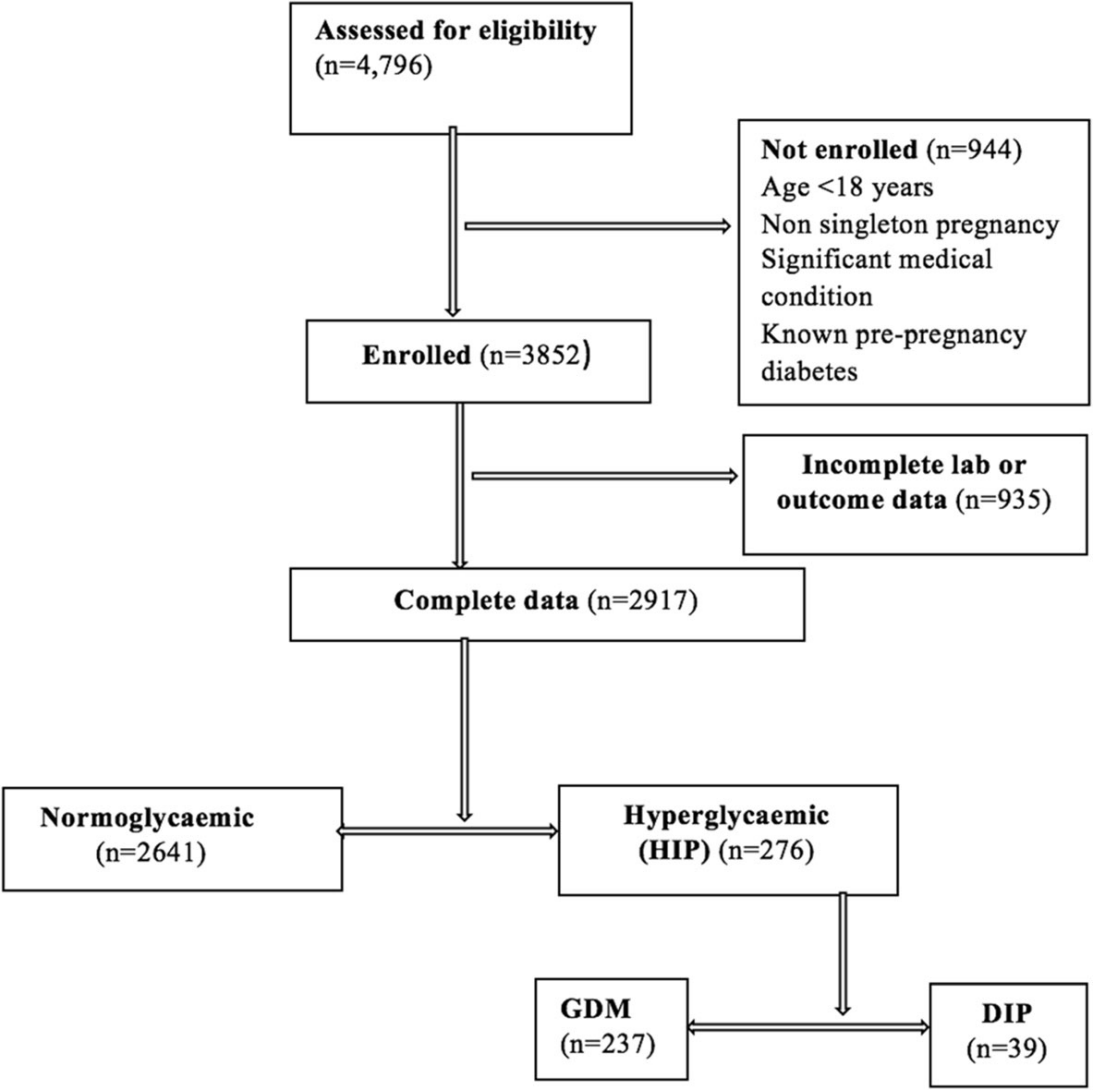
Flow of participants through the study

The study enrolled a total of 3852 participants. Of these, 2917 participants were included in the analysis. The remainder of the participants (*n* = 935) were excluded because they either had incomplete laboratory and/or outcome data (Fig. 1). Excluded participants had similar baseline characteristics as those included in the final analysis. Of those who were included in the analysis, 2641 were normoglycaemic and 276 had hyperglycaemia in pregnancy. Of these, 237 had hyperglycaemia in the GDM range, and 39 had hyperglycaemia in the DIP range.

The characteristics of participants are displayed in Table 1. All women in the study were of black ethnicity. The mean age of participants was 27.0 years, the mean maternal BMI at time of OGTT was 27.7 kg/m^2^. Nearly a third of women were primigravid (915/2917; 31.4%), around one third were primiparous (943/2917; 32.2%), and approximately one third were multiparous (two or more children: 1059/2917; 36.3%).

**Table 1:** Characteristics of study participants

	Normoglycaemic *n* = 2641 (Proportion) %	HIP *n* = 276		
			GDM *n* = 237	DIP *N* = 39
Study site				
Public %	54.2	38.4	39.7	30.8
Private %	45.8	61.6	60.3	69.2
Age	26.8 ± 5.4	28.9 ± 6.0	28.5 ± 6.0	31.7 ± 4.7
Mid-gestation BMI	27.5 ± 4.9	30.5 ± 6.3	30.4 ± 6.3	31.1 ± 5.9
Obese > 30	(696/2638) 26.4	(137/276) 49.6	(117/237) 49.4	(20/39) 51.3
Maternal height (cm)	158.7 ± 6.1	158.3 ± 5.9	158.3 ± 5.9	158.8 ± 5.8
HTN (> 140 mmHg or > 90 mmHg)	(26/2641) 1.0	(3/276) 1.5	(2/237) 0.8	(1/39) 2.6
MAP (mean)	80.1 ± 8.2	82.7 ± 8.8	82.2 ± 8.4	86.3 ± 10.7
HbA1c (Mean, DCCT)	4.8 ± 0.49	5.2 ± 0.79	5.1 ± 0.54	6.1 ± 1.43
Family history diabetes	(639/2437) 26.2	(91/258) 35.3	(74/222) 33.3	(17/36) 47.2
Prior macrosomia	(271/2641) 10.3	(48/276) 17.4	(39/237) 16.5	(9/39) 23.1
Current smoker	(3/2641) 0.1	(1/276) 0.4	(1/237) 0.4	(0/41) 0.0
Alcohol use in last 12 months	(476/2639) 18.0	(44/276) 15.9	(36/237) 15.2	(8/39) 20.5
Gravidity (mean)	2.5 ± 1.5	2.9 ± 1.7	2.9 ± 1.7	3.3 ± 1.7
Nulliparous	(852/2641) 32.3	(63/276) 22.8	(60/237) 25.3	(3/39) 7.7
Multiparous (≥ 2)	(935/2641) 35.4	(124/276) 44.9	(105/237) 44.3	(19/39) 48.7
HIV	(79/2641) 3.0	(15/276) 5.4	(10/237) 4.2	(5/39) 12.8

*BMI* body mass index, *HTN* hypertension in pregnancy, *MAP* mean arterial pressure, *HbA1c* glycated haemoglobin, *HIV* human immunodeficiency virus

Compared to normoglycaemic women, those with HIP were older, more likely to attend a private facility, and more likely to be obese, multiparous, and have a history of diabetes in the family or previous macrosomia. Compared to women with hyperglycaemia in the GDM range, those with DIP were more likely again to display these characteristics. In addition, those with DIP were more likely have a diagnosis of HIV.

### Antenatal management

The results of an audit detailing antenatal services available prior to the study are displayed in Table S1. The OGTT was not widely available and no sites used a defined screening or treatment protocol. Private facilities had more experience using oral hypoglycaemic agents and insulin. No site housed a joint clinic with physicians and obstetricians. Only private facilities ran a dedicated high-risk antenatal clinic for women with hyperglycaemia in pregnancy. No sites used a protocol for planning delivery or intrapartum glycaemic control. Equipment for capillary blood glucose (CBG) monitoring was available in private facilities, but generally unavailable in public facilities due to supply issues, and women were expected to provide their own. Neonatal services for treating complications such as prematurity and hypoglycaemia were generally available across all sites. No sites used protocols for managing women with diabetes during antenatal steroid use, sepsis, and post-partum.

Women diagnosed with HIP were seen on average twice in the diabetes antenatal clinic after their diagnosis (median, 2; IQR, 1–4). Approximately half of women with GDM (49.8%) received lifestyle advice only, 19.4% received low dose metformin (≤1 g/day), 1.7% received higher dose metformin (> 1 g/day) and one woman received insulin as a twice daily premixed preparation. Only one woman, who was treated with insulin, performed home self-monitoring of blood glucose. Women with DIP were more likely to be treated with metformin than the GDM group (48.7% Vs 21.1), and at a higher dose. No women with DIP were prescribed insulin. Data on management was missing for around one quarter of women with both GDM and DIP.

Half of the women with HIP (48.6%) had less than two fasting glucose measurements documented during their antenatal follow-up. Of those with GDM with more than two measurements, 26.2% appeared controlled (mean of 2 fasting blood glucose values < 5.1 mmol/L), 22.4% appeared partially controlled (mean of 2 fasting blood glucose values 5.1–7.0 mmol/L), and 1.7% seemed uncontrolled (mean of 2 fasting blood glucose values > 7.0 mmol/L). Those with DIP were less well controlled with 18.0% appearing uncontrolled during the antenatal period. Few women with GDM and DIP (15.6 and 12.8% respectively) had documentation of a third trimester ultrasound scan. A summary of the antenatal diabetes management of women with GDM and DIP is shown in Table 2.

**Table 2:** Antenatal diabetes management of women with GDM and DIP. ANC, antenatal clinic

	GDM *n* = 237	DIP *n* = 39
Diabetes ANC visits, Median (IQR)	2 (1–4)	2.4 (1–4)
**Treatment**		
Lifestyle advice only	(118/237) 49.8	(10/39) 25.6
Metformin ≤1 g/day	(46/237) 19.4	(14/39) 35.9
Metformin > 1 g/day	(4/237) 1.7	(5/39) 12.8
Insulin	(1/237) 0.4	(0/39) 0.0
Unknown	(68/237) 28.7	(10/39) 25.6
**Control**		
Controlled^a^	(62/237) 26.2	(7/39) 17.8
Partially controlled^b^	(53/237) 22.4	(9/39) 23.1
Uncontrolled^c^	(4/237) 1.7	(7/39) 18.0
Unknown	(118/237) 49.8	(16/39) 41.9
**Third trimester scan**		
Documented	(37/237) 15.6	(5/39) 12.8

^a^Mean of two fasting capillary glucose concentrations prior to delivery < 5.1 mmol/L

^b^Mean of two fasting capillary glucose concentrations prior to delivery 5.1–7.0 mmol/L

^c^Mean of two fasting capillary glucose concentrations prior to delivery > 7.1 mmol/L

### Maternal outcomes

Participants with HIP had higher risk of hypertensive disorders in pregnancy compared to the normoglycaemic participants (9.8% Vs 3.8%; *p*-value < 0.001). The proportion of those with hypertensive disorders was higher in the DIP group compared to the GDM group (20.5% Vs 8.0%). There was no difference in detection of poly−/oligohydramnios between the HIP and normoglycaemic groups. A higher proportion of women with HIP underwent Caesarean delivery as compared to normoglycaemic participants (38.4% Vs 28.7%; *p*-value < 0.001). Approximately three quarters (73.2%) of Caesarean deliveries were coded as ‘Emergency’ rather than ‘Elective’, with similar distribution among the HIP and normoglycaemic groups. There were no significant differences in other maternal delivery complications between the groups (Table 3). There was one maternal death, in the HIP group, due to intrapartum haemorrhage from a ruptured uterus secondary to obstructed labour.

**Table 3:** Maternal and neonatal delivery outcomes of normoglycaemic women, and those classified as GDM

	Normoglycaemic *n* = 2641 (Proportion) %	HIP *n* = 276	Risk Ratio (95% CI)	*p*-value		
					GDM *n* = 276	DIP *n* = 39
***MATERNAL OUTCOMES***						
Maternal Death	(0/2917) 0.0	(1/276) 0.003	–	–	(1/237) 0.004	(0/39) 0.0
Antepartum complications						
Hypertensive disorder	(101/2641) 3.8	(27/276) 9.8	2.56 (1.70–3.84)	< 0.001	(19/237) 8.0	(8/39) 20.5
Poly/oligohydramnios	(34/2641) 1.3	(7/276) 2.5	1.97 (0.88–4.40)	0.09	(7/237) 3.0	(0/39) 0.0
Caesarean delivery	(743/2591) 28.7	(104/271) 38.4	1.34 (1.14–1.57)	< 0.001	(93/234) 39.7	(11/37) 29.7
Maternal delivery complications						
Prolonged labour	(168/2641) 6.4	(16/276) 5.8	0.91 (0.55–1.50)	0.71	(15/237) 6.3	(1/39) 2.6
Ruptured uterus	(5/2641) 0.2	(0/276) 0.0	–	–	(1/237) 0.0	(0/39) 0.0
Shoulder dystocia	(3/2641) 0.1	(0/276) 0.0	–	–	(0/237) 0.0	(0/39) 0.0
***NEONATAL OUTCOMES***						
Perinatal mortality	(74/2641) 2.8	(7/276) 2.5	0.91 (0.42–1.95)	0.80	(5/237) 2.1	(2/39) 5.1
Stillbirth (> 24 weeks)	(45/2641) 1.7	(5/276) 1.8	1.06 (0.43–2.66)	0.90	(3/237) 1.3	(2/39) 5.1
Neonatal death (< 28 days)	(29/2567) 1.1	(2/271) 0.7	0.65 (0.16–2.72)	0.56	(2/233) 0.9	(0/37) 0.0
Mean birthweight (g)	3268.1 ± 551	3327.5 ± 535	–	0.10	3315.1 ± 512	3403.3 ± 660
LGA (>90th centile)	(514/2493) 20.6	(68/264) 25.8	1.30 (1.00–1.68)	0.05	(56/227) 24.7	(12/37) 32.4
Macrosomia (> 4 kg)	(212/2503) 8.5	(30/264) 11.4	1.34 (0.94–1.92)	0.11	(22/227) 9.7	(8/37) 21.6
SGA (<10th centile)	(235/2493) 9.4	(21/264) 8.0	0.84 (0.55–1.29)	0.43	(20/227) 8.8	(1/37) 2.7
Low birth weight (< 2.5 kg)	(211/2503) 8.4	(22/264) 8.3	0.99 (0.65–1.51)	0.96	(19/227) 8.4	(3/37) 8.1
Mean GA at delivery (weeks)	38.7 ± 1.8	38.5 ± 1.7	–	0.23	38.6 ± 1.5	37.9 ± 2.3
Very Preterm birth (<34w)	(53/2596) 2.0	(6/271) 2.2	1.08 (0.47–2.50)	0.85	(3/234) 1.3	(3/37) 8.1
Preterm birth (< 37 weeks)	(328/2596) 12.6	(38/271) 14.0	1.11 (0.81–1.52)	0.51	(29/234) 12.4	(9/37) 24.3
Neonatal admission	(289/2577) 11.2	(44/269) 16.4	1.45 (1.09–1.95)	0.01	(33/232) 14.2	(11/37) 29.7
Median length stay, IQR (days)	2 1–4	2 1–3	–		2 1–3	2.5 2–4
Umbilical cord c-peptide *n* = 445						
Median, IQR (mcg/L)	0.53 0.17–0.89	0.48 0.24–1.03	–	–	0.48 0.28–0.91	0.64 0.19–1.50
Neonatal complications						
Hypoglycaemia	(9/2588) 0.4	(4/263) 1.5	4.37 (1.36–14.1)	< 0.01	(4/227) 1.8	(0/36) 0.0
Jaundice	(48/2612) 1.8	(2/271) 0.7	0.40 (0.10–1.64)	0.19	(1/232) 0.4	(1/39) 2.6

*LGA* large for gestational age, *SGA* small for gestational age, *GA* gestational age, *IQR* interquartile range

### Neonatal outcomes

There was no significant difference detected in perinatal mortality rates between HIP and normoglycaemic pregnancies (2.5% Vs 2.8%; *p*-value 0.80). There was a difference detected in the proportion of babies born large for gestational age between the groups (25.8% Vs 20.6%; p-value 0.05). Babies born to mothers with HIP were on average 59.4 g larger than those born to normoglycaemic participants, but this was not statistically significant. No difference was detected in the proportion of babies born macrosomic, small for gestational age, low birthweight, premature or very premature between the two groups. More babies born to mothers with HIP were admitted to the neonatal unit with a similar length of stay to those babies admitted from normoglycaemic pregnancies. There was no difference in median umbilical cord c-peptide concentration between the groups, however there was a difference between babies with hypoglycaemia (1.5% Vs 0.4%; *p*-value < 0.01). There was no difference in proportion of babies with neonatal jaundice between the two groups.

As compared to GDM, those babies born to women with DIP appeared more likely to be large for gestational age, macrosomic, premature or very premature. Significance tests were not used due to the small numbers of women with DIP. Neonatal outcomes are displayed in Table 3.

## Discussion

### Main findings

Our study in Ugandan women shows that HIP is a common, poorly managed condition associated with a high frequency of adverse pregnancy outcomes. These included large for gestational age infants, Caesarean delivery and neonatal admission, but not preterm birth or perinatal death. These adverse events were mostly seen in women with DIP, with frequency of poor outcomes in women with GDM similar to those women with normoglycaemic pregnancies.

### Interpretation (in light of other evidence)

Sub-Saharan Africa is undergoing a rapid demographic and nutritional transition. With an emerging epidemic of type 2 diabetes across the continent, hyperglycaemia in pregnancy is likely to pose a major health challenge in this region in the future.

Our study demonstrated that the management of women with HIP was not intensive, with approximately one quarter of women receiving metformin, and only one (of the 276) treated with insulin to control glycaemia. Moreover, in most women, fasting glucose measurements were done only 1–4 times during the entire course of their third trimester. Similarly, very few women had a third trimester growth scan which is usually a hallmark of diabetes antenatal management and delivery planning. It is likely that these deficiencies in practice compromise glycaemic control and contribute to increased risk of complications. Nonetheless, the clinical management of participants within the study environment is likely to have been more intensive than usual practice; our audit prior to the study highlighted deficiencies in usual care, particularly the disparity in services available at public and private facilities.

Our data show that HIP was associated with significantly increased risk of large birthweight offspring. In addition, GDM was associated with increased likelihood of operative delivery. It was not documented whether the Caesarean deliveries were primary or repeat procedures from other previous complications. Nonetheless, it is likely that macrosomia was contributory, particularly since women in sub-Saharan Africa are already more prone to obstructed labour due to cephalopelvic disproportion [12–15].

We did not observe any association between HIP and perinatal mortality. This may, in part, be because the study was not powered for this outcome. We observed few perinatal deaths; the background rate of perinatal mortality in Uganda 41 per 1000 births (eight times greater than the HAPO study [16, 17]), but we observed a lower than expected figure of 28 per 1000 births – perhaps because our study was biased towards the urban population. However, HIP was associated with an increased risk of neonatal admission and neonatal hypoglycaemia. Whilst this outcome is limited by the predilection to test babies born to mothers with an unblinded diagnosis of HIP, the paucity of glucose testing reflects the challenges faced in this setting to detect neonatal hypoglycaemia. This offers a potential argument in favour of testing and managing HIP, however no reduction in neonatal hypoglycaemia was detected in either of the large trials assessing treatment of GDM, even in the context of management strategies that would nonetheless be unfeasible in our setting [4, 5].

Almost all pregnancy outcomes were worse for women identified as DIP rather than GDM. Although significance tests were not performed for these individual sub-groups (due to small sample size), it is likely that differences between the HIP and normoglycaemic groups may be driven by the adverse pregnancy outcomes suffered by women with overt diabetes in pregnancy, rather than GDM. When the latest international diagnostic cut-off values are employed, the prevalence of GDM appears to be high (Natamba et al. unpublished). Therefore, where resources are limited, screening and management should be targeted to those women with DIP who appear more at risk. To give some context, the per capita health expenditure in Uganda is $37.6 per year, compared to $3958 per year in the UK (2016 figures) [18]. Although there are no cost-effectiveness studies for HIP screening from Africa, a study from India estimated a cost of $1626 per life year gained [19]. Furthermore, costs of management, which may include insulin therapy and ambulatory capillary glucose monitoring, are absorbed by women and are currently prohibitively expensive to most [20]. This local evidence is therefore crucial to ensure that only women most at risk of clinically relevant adverse pregnancy outcomes are identified and treated. More work is needed to delineate which women should be tested for hyperglycaemia, by what method, and how their needs are best met at health facilities. Our baseline audit clearly demonstrates areas for potential improvement. Some deficiencies in care are clearly dependent on the cost of testing and treatment materials, and may be difficult to address immediately in light of other important competing interests. However, organisation of antenatal services to ensure women at high risk of pregnancy complications are seen in dedicated clinics is not only achievable but may have great impact.

### Strengths and limitations

To our knowledge this is the largest and most robust prospective investigation of pregnancy outcomes in HIP in sub-Saharan Africa. We made efforts to ensure that it is as representative as possible by recruiting from both public and private facilities. We made use of a rigid study protocol to collect detailed data, utilised high quality central laboratory for sample analyses, employed up-to-date diagnostic criteria.

In our study, women diagnosed with GDM were referred for management by their clinicians, rather than through a study protocol; the intensity of treatment was therefore likely to be variable and not under study control. Also, women with HIP may have received better overall antenatal care due to frequency of study visits; this may reduce the frequency of poor outcomes compared to the situation when HIP is not detected, as is usual without screening in the general population. Our primary outcome, birthweight centile, is calculated using gestational age at delivery, which was calculated using either early obstetric ultrasound scan or last menstrual cycle at booking. Estimation of gestational age is notoriously difficult in this setting, but this would have been true in women with hyperglycaemia in pregnancy as well as those without. For some of the outcomes (such as pre-eclampsia, poly/oligohydramnios) we relied on healthcare records rather than active investigation by the study team. We did not have data regarding previous operative delivery and so could only report Caesarean delivery, rather than primary Caesarean delivery. The management of hyperglycaemia was not standardised, and our assessment was based on a small amount of data on fasting capillary blood glucose levels; this does not take into account post-prandial glycaemic variability, which can make an important contribution to glycaemic burden and complications. The study was performed in urban and peri-urban central Uganda which may reduce generalisability to rural populations.

## Conclusion

Our data from Uganda indicate that HIP is associated with adverse pregnancy outcomes, particularly for women with overt diabetes in pregnancy. Those with hyperglycaemia in the GDM-range appeared more modestly affected, despite suboptimal third trimester diabetes antenatal management. Screening and management strategies should be targeted to women with overt diabetes in pregnancy who appear most at risk of adverse pregnancy outcomes. More studies are needed to understand the independent contribution of glucose levels and other non-glucose physiological factors such as obesity and hypertension, and the effect of HIP beyond immediate peripartum complications such as fetal programming which may increase lifetime cardiovascular risk for both mother and baby [21, 22].

## Supplementary Information

**Additional file 1: Table S1.** Summary of diabetes antenatal services available at five study sites prior to commencement of the study.

## Data Availability

The datasets used and/or analysed during the current study are available from the corresponding author on reasonable request.
