# Supplementary material for: Antenatal management and maternal/fetal outcomes associated with hyperglycaemia in pregnancy (HIP) in Uganda; a prospective cohort study
Source: BMC Pregnancy Childbirth. 2021 May 19;21:386. doi: 10.1186/s12884-021-03795-5 (PMC8132348; doi:10.1186/s12884-021-03795-5)
Supplement: Supplementary file 1 — Additional file 1: Table S1. Summary of diabetes antenatal services available at five study sites prior to commencement of the study. [file 12884_2021_3795_MOESM1_ESM.docx]

*Title Page for Table S1*

**Antenatal management and maternal/fetal outcomes associated with hyperglycaemia in pregnancy (HIP) in Uganda; a prospective cohort study**

Jack Milln (MBBS)^1,2^* and Betty Nakabuye (MMED)^3,4^, Barnabas Kahiira Natamba (PhD)^1^, Isaac Sekitoleko (MSc)^1^, Michael Mubiru (BSc)^1^, Arthur Araali Namara (MSc)^1^, Samuel Tumwesigire (MBchB)^1^, Salome Tino (MPH)^1^, Mandy Mirembe (MSc)^1^, Ayoub Kakande (MSc)^1^, Brian Agaba (MMED)^3^, Faridah Nansubuga (MMED)^5^, Daniel Zaake (MMED)^5^, Ben Ayiko (MMED)^6^, Herbert Kalema (MMED)^7^, Sarah Nakubulwa (PhD)^8,9^, Musa Sekikubo (PhD)^8,9^, Annettee Nakimuli (PhD)^8,9^, Emily L Webb (PhD)^10^, Moffat J Nyirenda (PhD)^1,10^

^1^ Non-Communicable Diseases Theme, Medical Research Council/Uganda Virus Research Institute and London School of Hygiene and Tropical Medicine (MRC/UVRI & LSHTM) Uganda Research Unit, Plot 51-59, Nakiwogo Road, P. O. BOX 49, Entebbe, Uganda.

^2^ Department of Endocrinology and Diabetes, Queen Mary University of London, Mile End Road, London, UK.

^3^ Rubaga Uganda Martyrs Hospital, Kampala, Uganda.

^4^ School of Public Health, Makerere University College of Health Sciences, Kampala, Uganda

^5^ St. Francis Hospital, Nsambya, Kampala, Uganda

^6^ Entebbe Regional Referral Hospital, Entebbe, Uganda

^7^ Masaka Regional Referral Hospital, Masaka, Uganda

^8^ Department of Obstetrics and Gynaecology, School of Medicine, Makerere University College of Health Sciences, Kampala, Uganda

^9^ Kawempe National Referral Hospital, Kampala, Uganda

^10^ London School of Hygiene and Tropical Medicine (LSHTM), London, UK

* Corresponding author

**Corresponding author:**

Dr Jack Milln

MRC/UVRI & LSHTM Uganda Research Unit, Plot 51-59, Nakiwogo Road, P. O. BOX 49, Entebbe, Uganda.

Tel.: +256 793 392872. No fax available

[jackmilln@doctors.org.uk](mailto:jackmilln@doctors.org.uk)

**Table S1:**

**
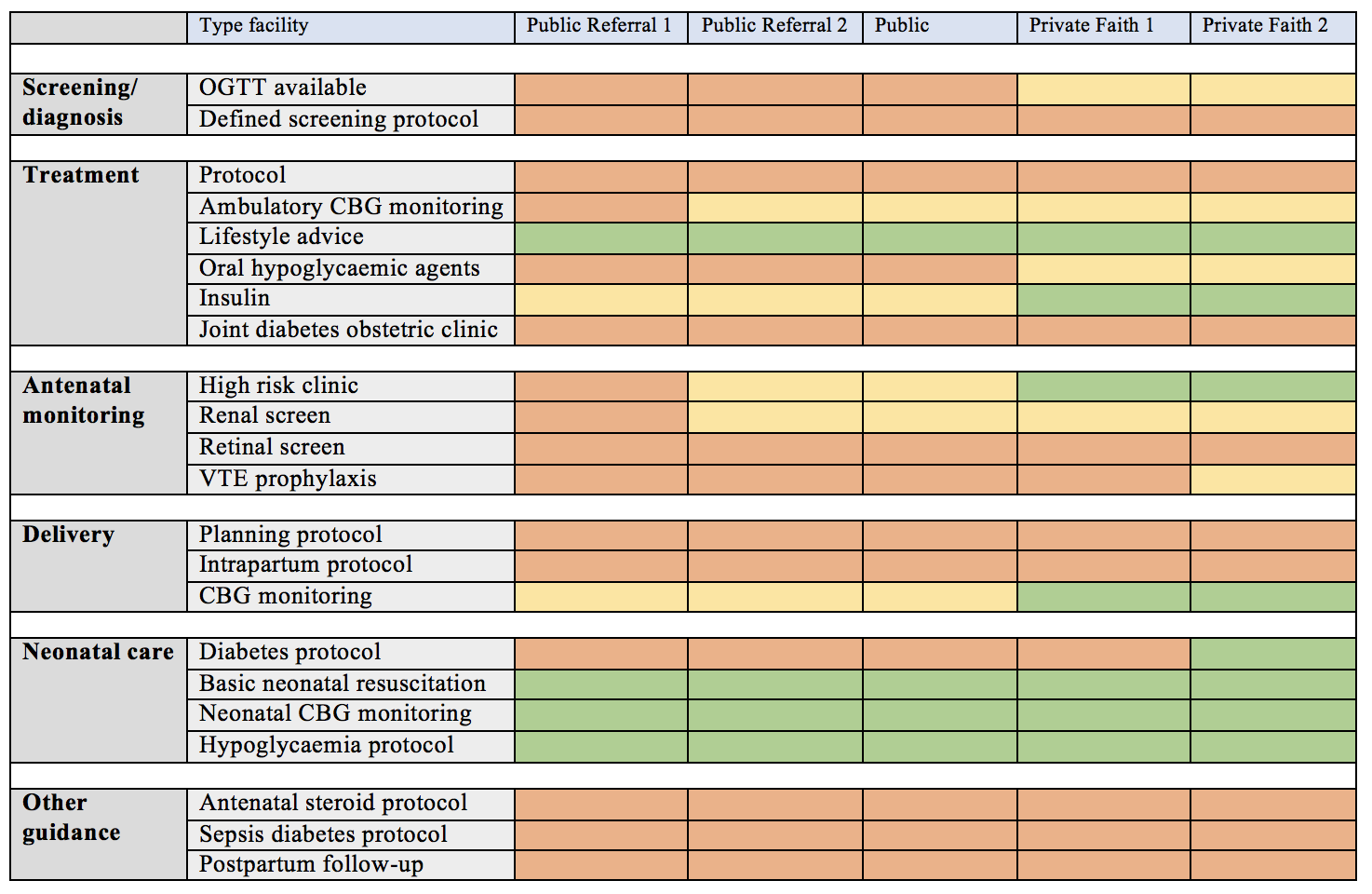
**

**Table S1:** Summary of diabetes antenatal management at five study sites prior to commencement of the study. Red, absent at the facility; yellow, occasionally done but without documentation of guidelines; green, regularly done and documentation of guidelines present. CBG, capillary blood glucose.
